# Supplementary material for: Real-time identification of epistatic interactions in SARS-CoV-2 from large genome collections
Source: Genome Biol. 2024 Aug 22;25:228. doi: 10.1186/s13059-024-03355-y (PMC11342480; doi:10.1186/s13059-024-03355-y)
Supplement: Supplementary file 6 — Additional file 6. Supplementary figures S1-11. [file 13059_2024_3355_MOESM6_ESM.docx]

## **Supplementary Figures**

**Figure S1.** **Influence of dataset size on mutual information estimation, using only O3 and O4 interactions.**
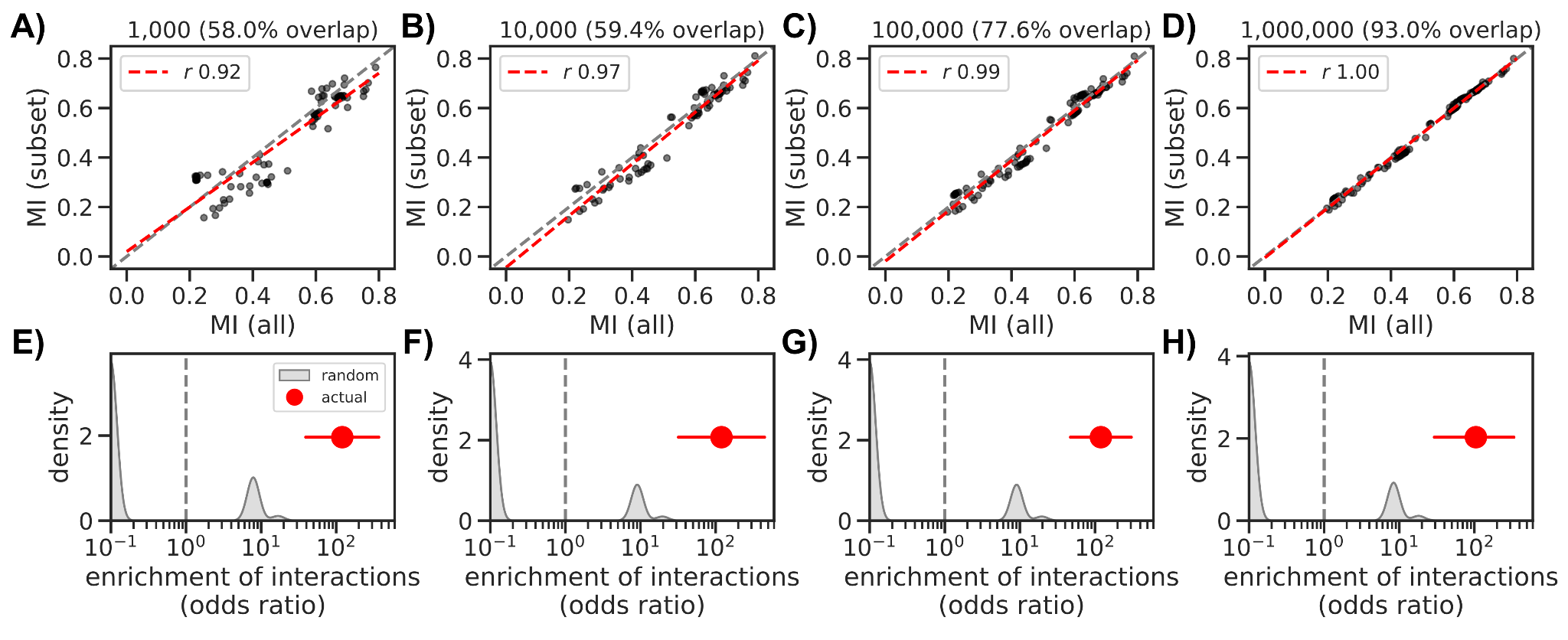
(A-D) Linear regression analysis of the intersection of mutual information values between interactions belonging to Outlier level 3 and 4 in the whole dataset versus the same Outliers in reduced datasets of (A) 1000, (B) 10,000, (C) 100,000 and (D) 1,000,000 sequence subsets. (E-H) Enrichment of interactions between Outliers 3 and 4 positions known to epistatically interact (vertical red line) versus a series (N=1,000) of random RBD networks with the same number of interactions as the real one (gray distribution). Subsets are the same as the panel directly above: 1,000 (F), 10,000 (G), 100,000 (H), and 1,000,000 (I).

**Figure S2: Representation of the Hill curve function that was used for the time weighting.**
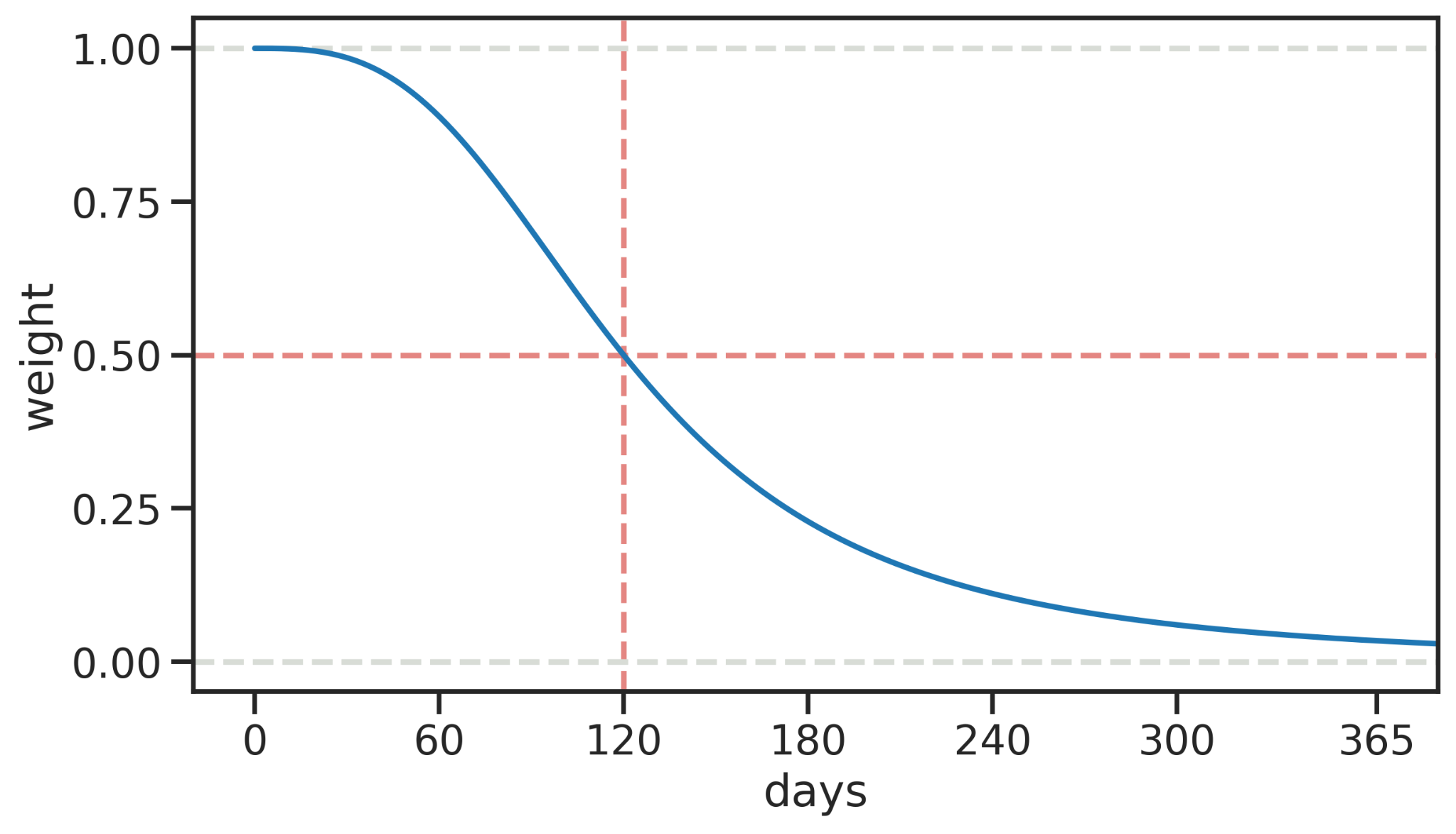
 The Hill coefficient was set to 3, and the function allowed a reduction of 0.5 of the initial sequence weight (weight=1 at the focal time point) after 120 days (~4 months).


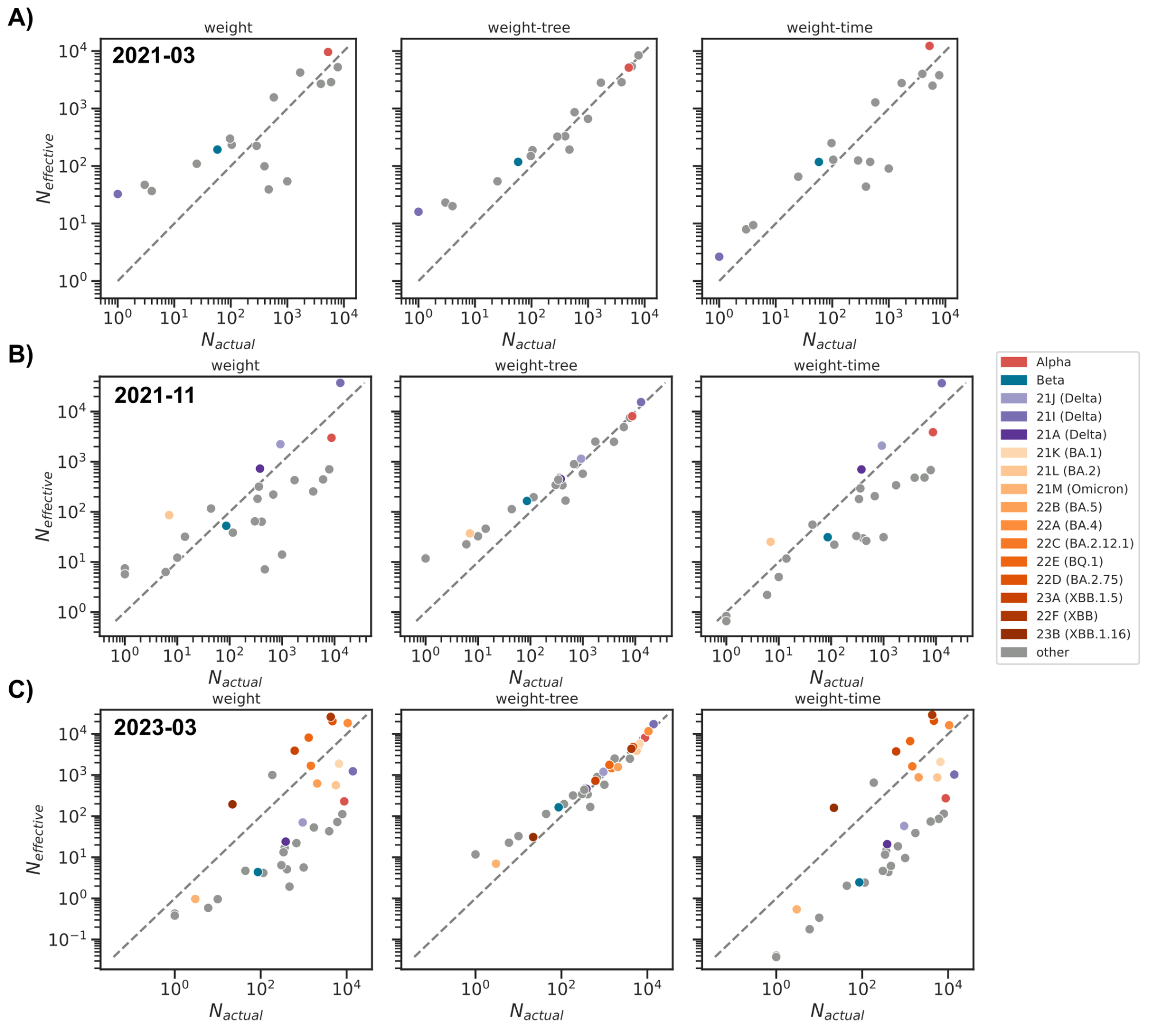


**Figure S3: Comparison between number of actual samples belonging to each lineage versus its effective number according to the weighting scheme used to compute MI values.** The final weight (left), and the phylogeny-based (center) and time-based (right) components are shown for three time points: 2021-03 (A), 2021-11 (B), and 2023-03 (C) are shown..


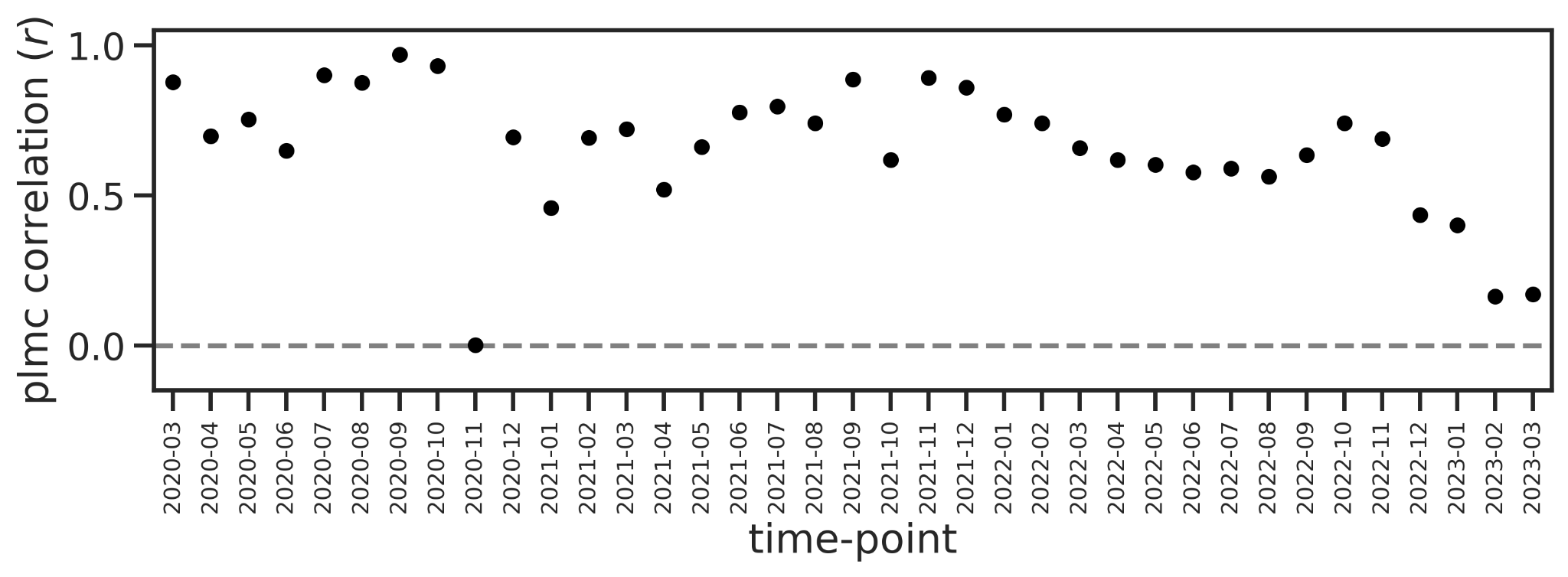


**Figure S4.** **Correlation between mutual information and the plmc implementation of the DCA method.** For each time point, the Pearson’s correlation coefficient *r* between the MI and plmc values is reported.


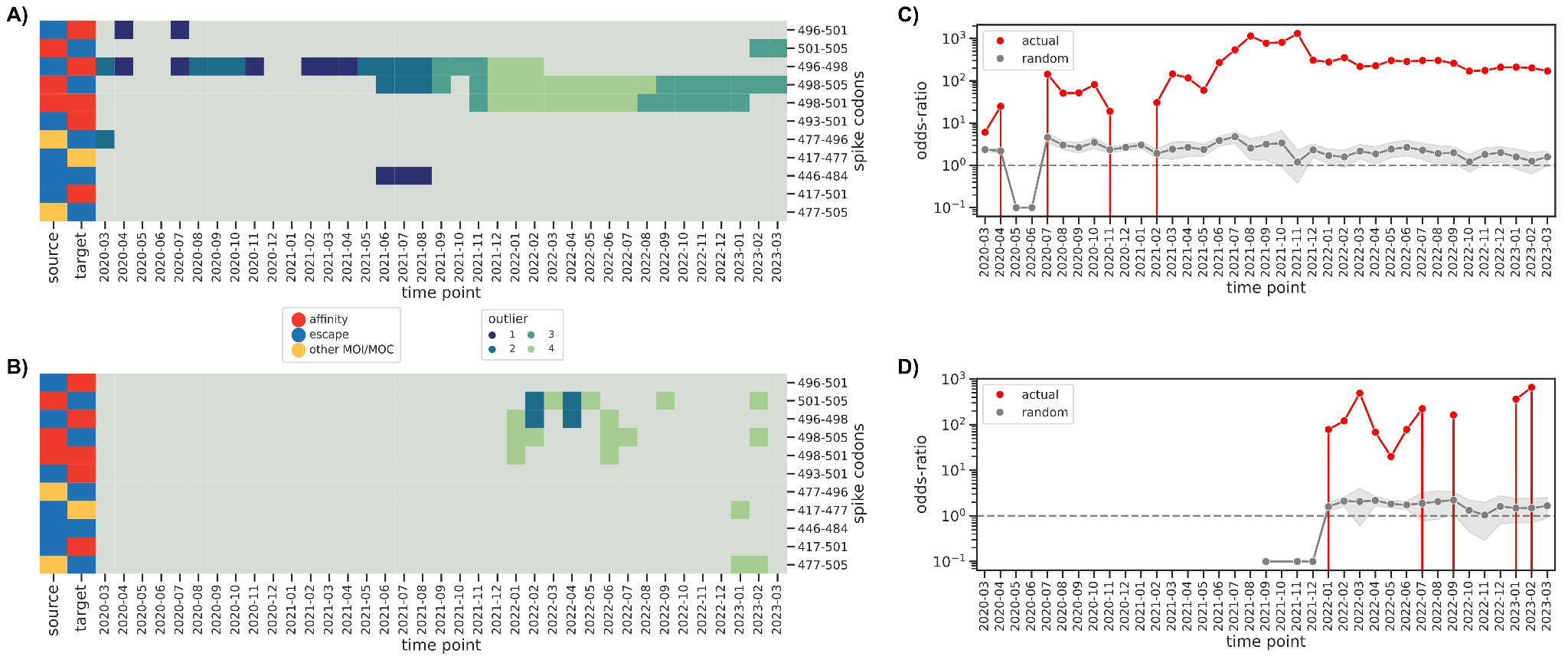


**Figure S5. Comparison between the mutual information and the DCA based methods to retrieve known epistatic interactions. Top row shows the results for the MI method, bottom DCA.** A) and B) Presence/absence matrix of predicted interactions between Spike codons with experimentally validated epistatic interactions. Gray indicates no predicted interaction. C) and D) Enrichment of interactions between positions interacting epistatically (red dots) versus a series (N=1,000) of random RBD networks with the same number of interactions as the real one.

**Figure S6.** **Alternative validation using known RBD epistatic interactions.**
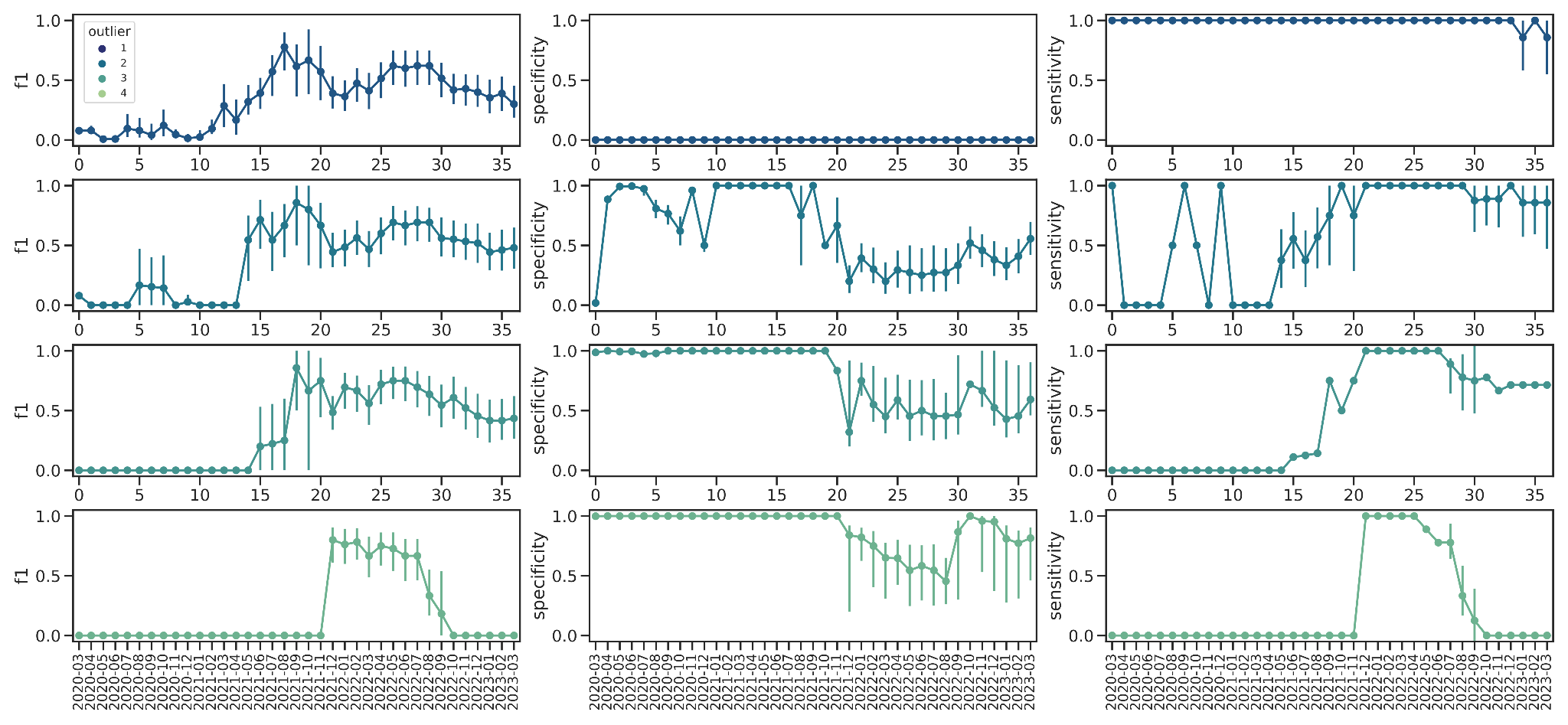
Performance of a binary classifier for RBD interactions using the four outlier thresholds (rows) and measuring three different indicators: F1 score (first column), specificity (second column), and sensitivity (third column). Vertical solid lines indicate the 95% confidence interval.

**Figure S7.** **Validation using predicted interactions before filtering through the ARACNE algorithm.**
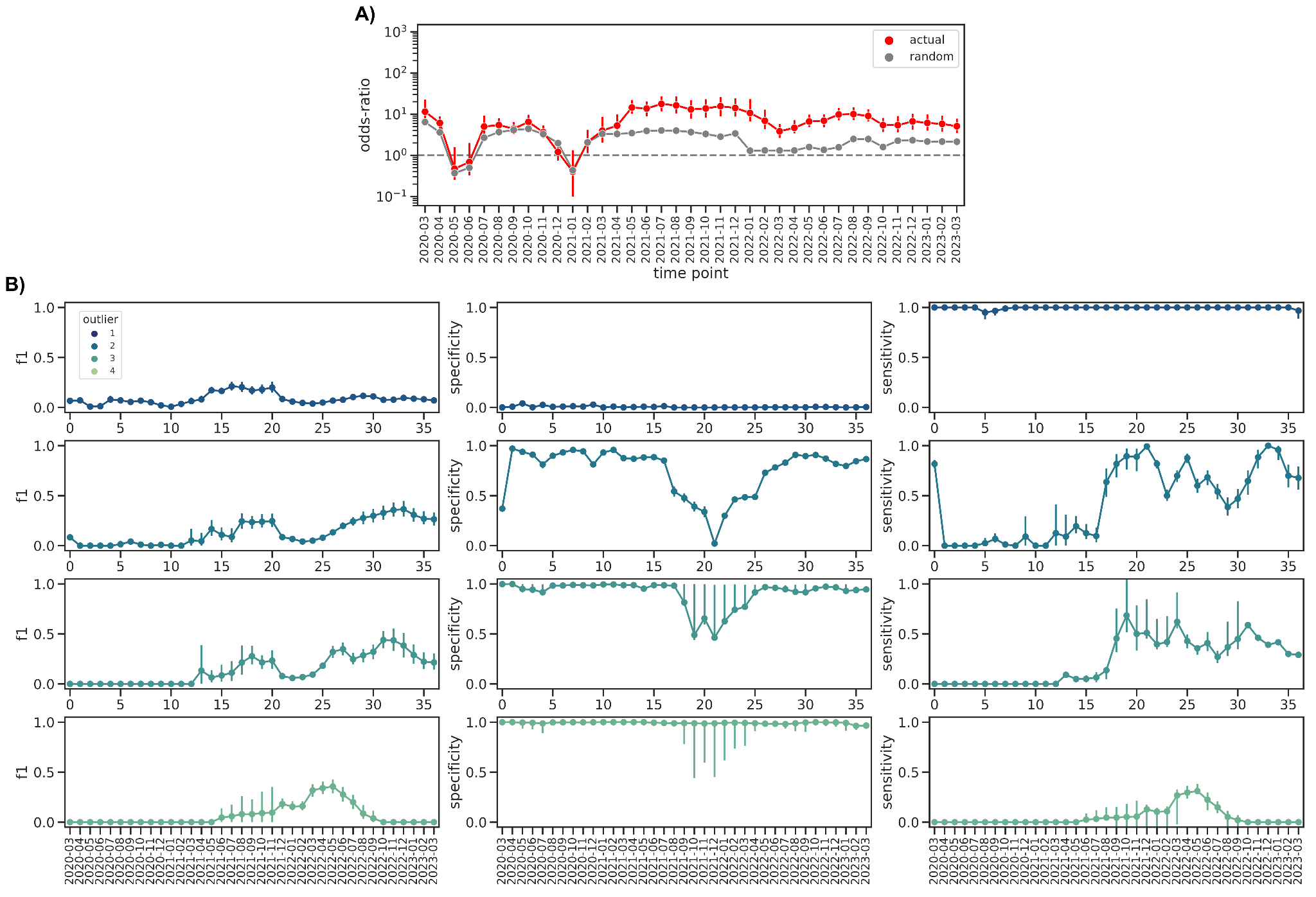
A) Enrichment of known interactions for the actual predictions (red) and for 1,000 random interaction networks (grey). Vertical red lines indicate the 95% confidence interval, grey shaded area the standard deviation. B) Performance of a binary classifier for RBD interactions using the four outlier thresholds (rows) and measuring three different indicators: F1 score (first column), specificity (second column), and sensitivity (third column). Vertical solid lines indicate the 95% confidence interval.


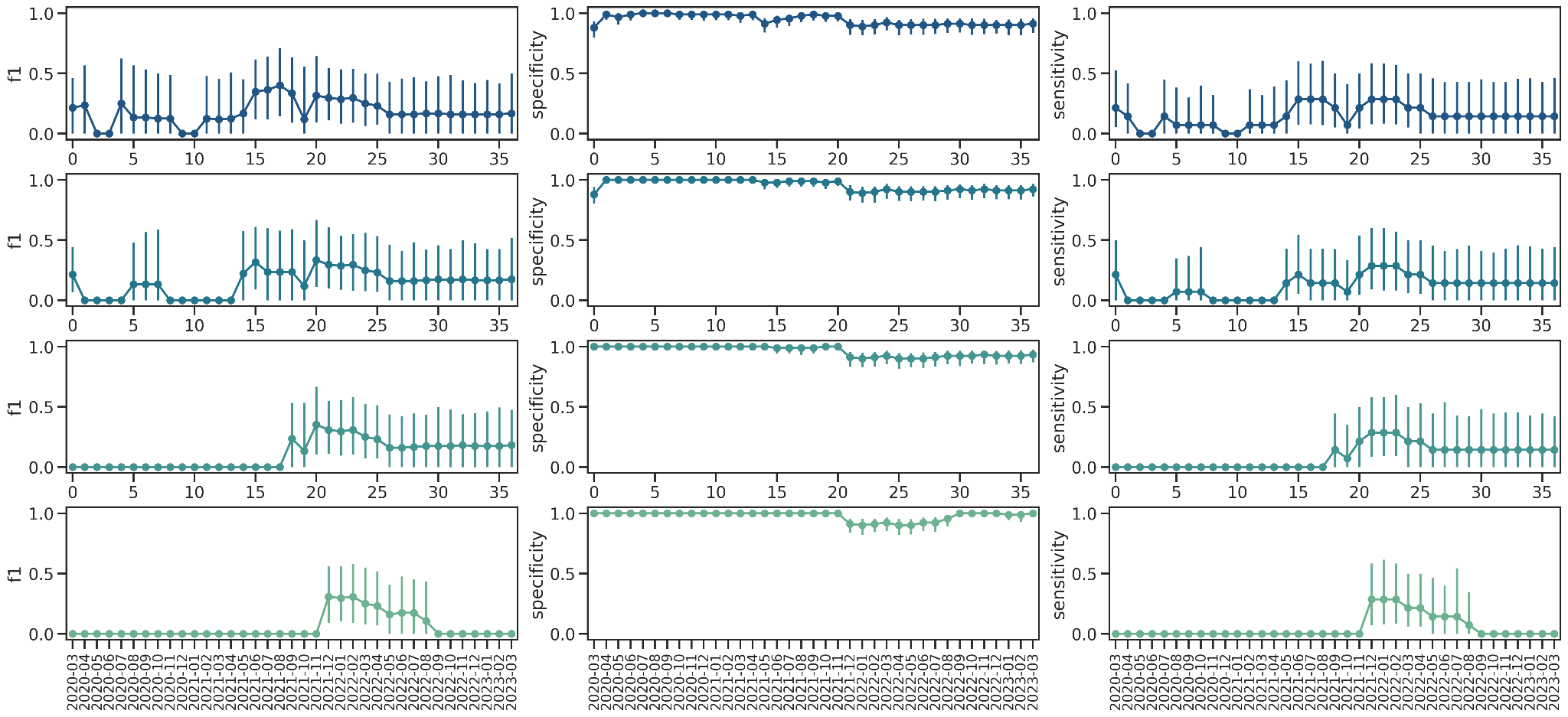


**Figure S8.** **Alternative validation using pairwise coefficients for 15 BA.1 mutations.** Performance of a binary classifier for RBD interactions using the four outlier thresholds (rows) and measuring three different indicators: F1 score (first column), specificity (second column), and sensitivity (third column). Vertical solid lines indicate the 95% confidence interval.

**Figure S9: Presence/Absence matrix of notable spike positions using the raw MI interaction data before the filtering with the ARACNE algorithm.**
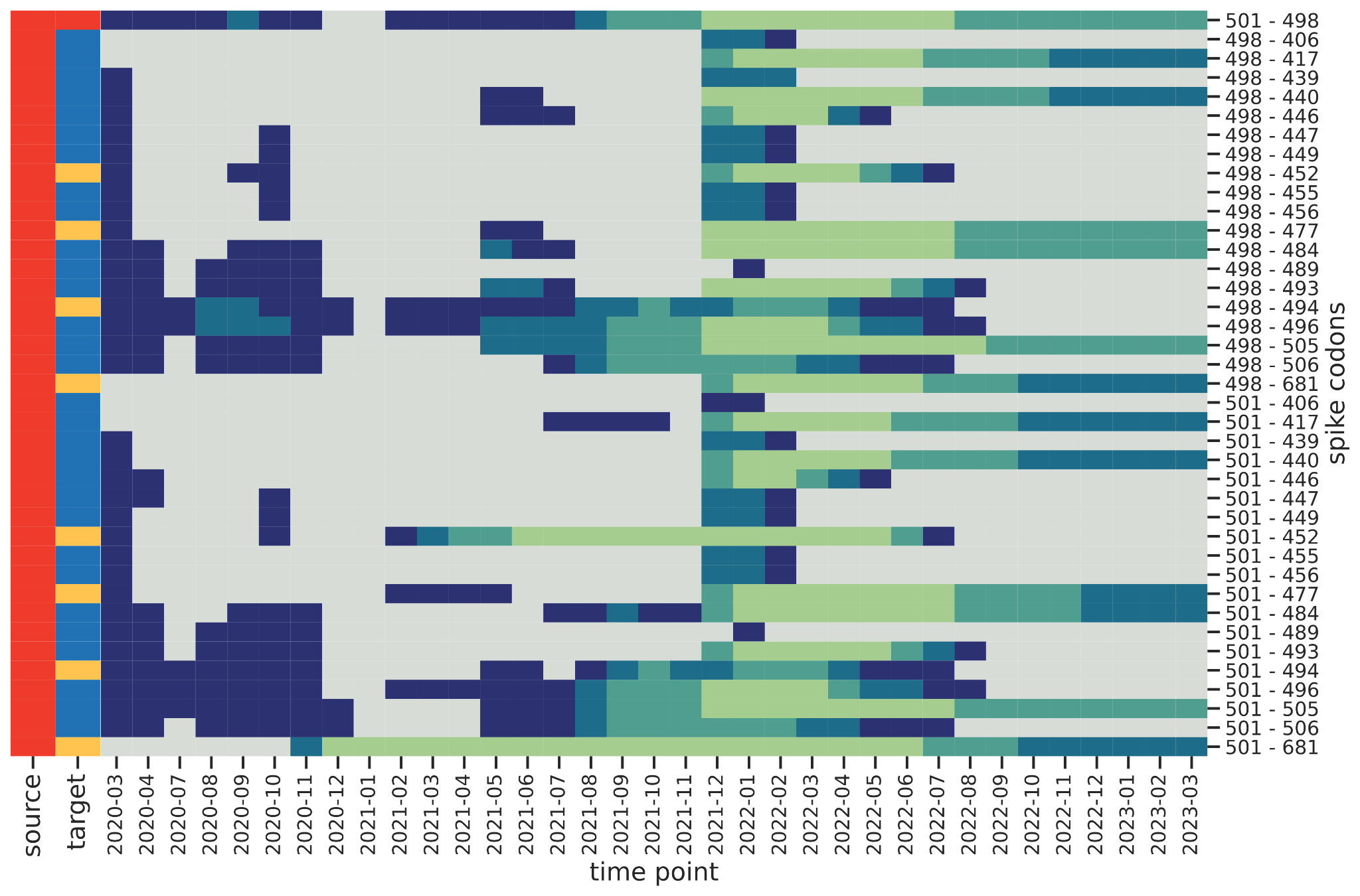
 Gray indicates absence of predicted interactions.


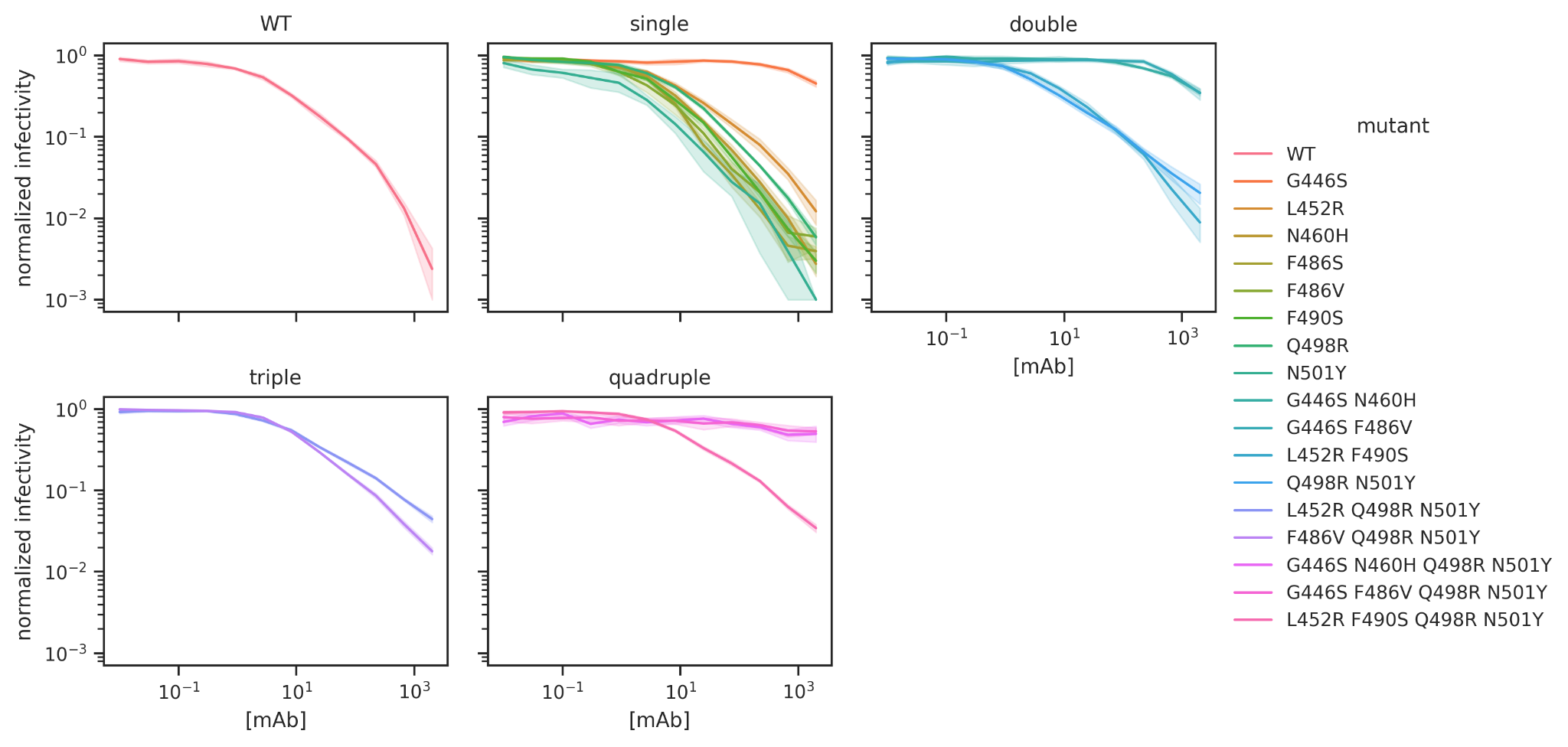


**Figure S10: Neutralization curves for each pseudovirus tested in this study.** Solid line indicates the mean over three replicates, shaded area the 95% confidence interval.

**Figure S11. Predicted epistatic interaction in the Spike gene as a function of time, before the removal of sequences with incorrect dating.**
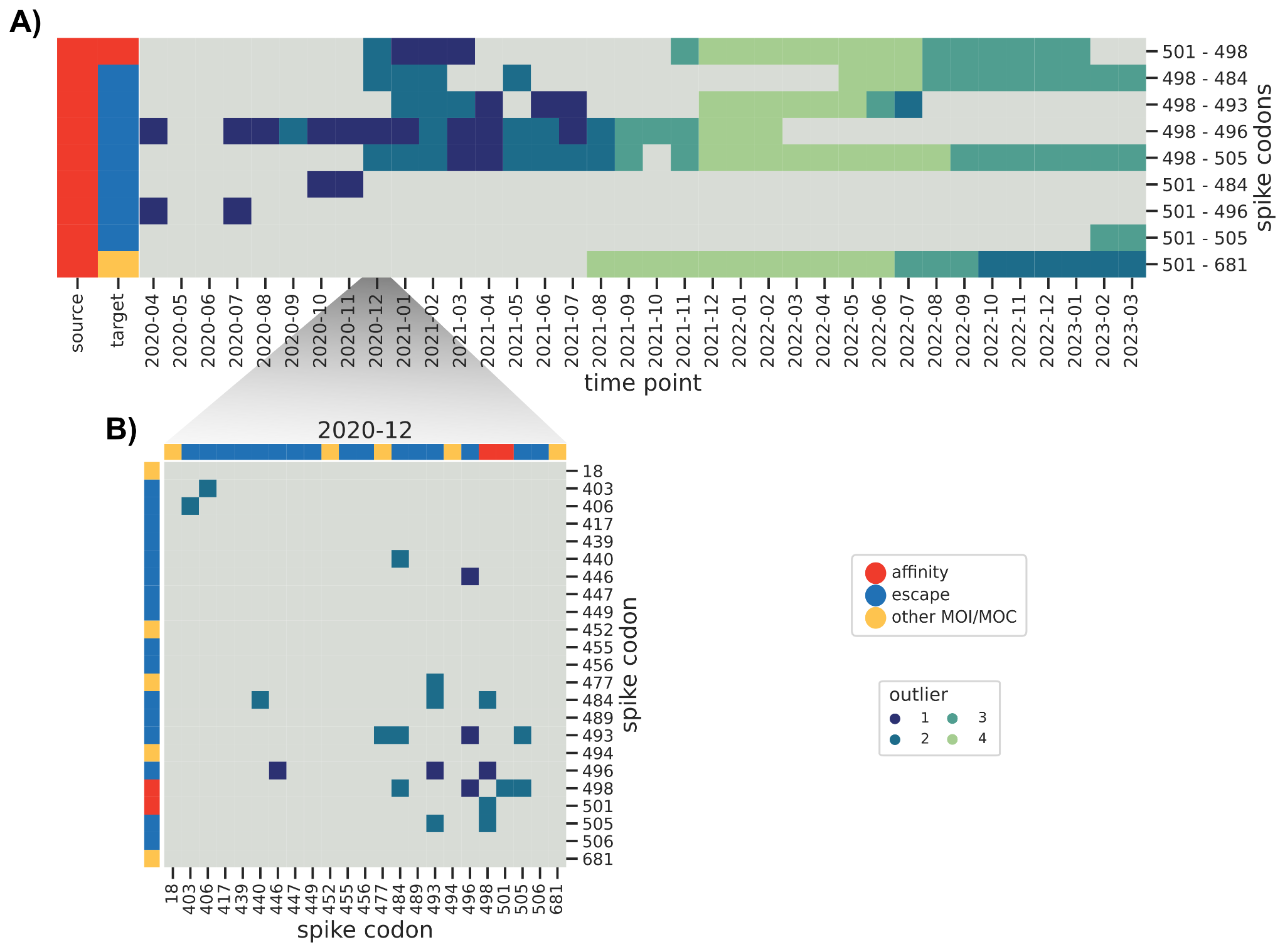
A) Presence/absence matrix of predicted interactions between Spike codons 498/501 and those labeled either as escape variants or other Mutation of Interest/Concern. Gray indicates no predicted interaction. B) Interaction heatmaps between selected Spike gene codons for December 2020.
