# Supplementary material for: Real-time identification of epistatic interactions in SARS-CoV-2 from large genome collections
Source: Genome Biol. 2024 Aug 22;25:228. doi: 10.1186/s13059-024-03355-y (PMC11342480; doi:10.1186/s13059-024-03355-y)
Supplement: Supplementary file 13 — Additional file 13. Review history. [file 13059_2024_3355_MOESM13_ESM.docx]

**Genome Biology review history**

**Reviewer 1**

The revised version of the manuscript has been improved greatly, but I still have two small comments as follows:

1. In the revised version, the epistasis inferred from MI method is compared with that from DCA method. However, as far as I know, with the implementations of DCA, some non-various loci even in the coding region (between 5' and 3' ends) could be filtered as the significance of the inferred epistasis including them could be very low. But the authors only mentioned the sequence filtering instead of that on the coding loci. Is it helpful for improvement of the inference efficiency?

2. With the solid theoretical support and wide applications of DCA methods in the biological /genomic dataset, it might be difficult to stimulate outstanding interest to a broad audience of biologists for the MI approach. It could be interesting to identify the significant dependencies by MI, and then use the DCA analyze their causality, to provide a more comprehensive understanding on the epistasis inference.

**Authors’ response**

Reviewer #1: The revised version of the manuscript has been improved greatly, but I still have

two small comments as follows:

1. In the revised version, the epistasis inferred from MI method is compared with that from DCA

method. However, as far as I know, with the implementations of DCA, some non-various loci

even in the coding region (between 5' and 3' ends) could be filtered as the significance of the

inferred epistasis including them could be very low. But the authors only mentioned the

sequence filtering instead of that on the coding loci. Is it helpful for improvement of the inference

efficiency?

We thank the reviewer for pointing out this strategy to reduce the computational requirements of

DCA-based methods. Removing positions with low diversity can indeed reduce the memory

required to load and process the input alignment, as well as reducing the computing time. We

have verified the impact of this kind of filtering by removing positions for which the combined

minimum allele frequency (MAF) of all alternative alleles was below a predefined threshold. We

applied these filters to the Spike alignment of the last time point (March 2023, ~93k sequences)

and measured the peak RAM usage and walltime of running plmc using 48 cores.. We observed

the following results:

| MAF threshold | Positions | Walltime (hrs) | Peak RAM (Gb) |
| --- | --- | --- | --- |
| N/A | 3822 | 4.5 | 9.67 |
| 0.0001 | 3686 | 4.5 | 9.04 |
| 0.001 | 2136 | 4.5 | 3.39 |
| 0.01 | 1080 | 0.5 | 0.8 |
| 0.05 | 145 | 0.2 | 0.48 |
| 0.1 | 109 | 0.2 | 0.48 |

While it is indeed possible to dramatically reduce the memory and time requirements of the plmc

implementation of DCA, we note that in order to be able to identify early signals of potential

epistatic interactions, we would need to use a MAF threshold not higher than 0.0001, which has

very similar computing requirements as the unfiltered dataset. This would imply that positions for

which less than 9 samples have an alternative allele would be filtered out. As we have shown in

the manuscript, the MI-based method identified a cornerstone epistatic interaction when just 7

samples encoding the double mutation were present in the sample. We do acknowledge that

there could be a trade-off between accuracy and sensitivity though.

**Action taken:** we have added a comment in the discussion section indicating how filtering the

input alignment might be a suitable strategy to make DCA estimation working on large datasets (“Filtering out positions with low diversity from the multiple sequence alignment might be a

relatively simple strategy to reduce the computational requirement of DCA-based methods,

although they could reduce their sensitivity, as emerging variants may be filtered out.”).

2. With the solid theoretical support and wide applications of DCA methods in the biological

/genomic dataset, it might be difficult to stimulate outstanding interest to a broad audience of

biologists for the MI approach. It could be interesting to identify the significant dependencies by

MI, and then use the DCA analyze their causality, to provide a more comprehensive

understanding on the epistasis inference.

We thank the reviewer for this suggestion. While we agree that developing such a two-step

system might help reduce the number of false positives, we believe that in practice finding the

right implementation details falls beyond the scope of our manuscript.

**Action taken:** we have added a comment in the discussion section (“We also note that

interacting pairs estimated with a method based on mutual information could then be further

filtered using a DCA-based method, which could reduce the false positive rate and simplify the

dense parts of the interaction graph; the theoretical and technical details of such a two-step

approach are however still to be properly defined.”), which hopefully will call the research

community into action to implement such an idea.
